# Thrombus in Transit on Point-of-Care Ultrasound in COVID-19 Pneumonia: A Cause of Refractory Hypoxia Requiring Systemic Thrombolysis

**DOI:** 10.7759/cureus.15599

**Published:** 2021-06-11

**Authors:** Neema Jayachamarajapura Onkarmurthy, Ibrahim Omore, Michelle Thomas, Farbod Raiszadeh

**Affiliations:** 1 Internal Medicine, Harlem Hospital Center, New York, USA; 2 Cardiology, Harlem Hospital Center, New York, USA

**Keywords:** thrombolysis, covid-19, severe acute respiratory syndrome coronavirus 2, sars-cov-2, pneumonia, deep vein thrombosis, point of care ultrasound, case report

## Abstract

Coronavirus disease 2019 (COVID-19) is predominantly a pulmonary disease due to infection with severe acute respiratory syndrome coronavirus 2 (SARS-CoV-2) with underlying systemic involvement associated with coagulopathy. The reported number of events of venous thromboembolism and refractory hypoxia remains high despite being maintained on prophylactic or therapeutic doses of anticoagulation in patients with a high clinical indication, which has shown a reduction in mortality otherwise.

This report is of a case of severe COVID-19 pneumonia in a 37-year-old Hispanic man who developed coagulopathy with left popliteal vein thrombosis and subsequently a right ventricle thrombus in transit diagnosed by point-of-care ultrasound requiring systemic thrombolysis.

Although patients with severe COVID-19 pneumonia are routinely given therapeutic anticoagulants, this case has shown that monitoring acute thrombotic events, D-dimer levels, and the presence of refractory hypoxia may indicate a thrombotic event that requires further intervention. This report has demonstrated the value of point-of-care ultrasound in the diagnosis of thromboembolism and venous thrombosis in a patient with severe COVID-19 pneumonia.

## Introduction

Coronavirus disease 2019 (COVID-19) patients requiring hospitalization are most commonly presenting with respiratory distress [1]. These patients with acute respiratory failure are noted to have a hypercoagulable state with elevated D-dimer levels due to excessive fibrin formation and polymerization that may predispose to thrombosis associated with refractory hypoxia and poor outcomes [2]. At the beginning of the pandemic, fewer cases were reported globally with venous thromboembolism (VTE) in COVID-19 [3,4]. However, the notable pattern of numerous venous thromboembolic events reported calls for being on the lookout, which would enable timely appropriate management [5-7]. The reported number of events with VTE and refractory hypoxia remains high [8] despite being maintained on prophylactic or therapeutic doses of anticoagulation in patients with a high clinical indication, which has shown to reduce mortality otherwise [9]. We present the case of a 37-year-old young man who came in with severe hypoxic respiratory failure due to COVID-19 pneumonia placed on therapeutic anticoagulation for elevated D-dimer with a confirmed left popliteal vein thrombosis who subsequently developed persistent refractory hypoxia, with a thrombus in transit detected in the right ventricle by point-of-care ultrasound (POCUS).

This article was previously presented as a finalist virtual e-poster at the National Abstract Competition - American College of Physicians in April 2021.

## Case presentation

A 37-year-old Hispanic male with no known past medical history presented with a three-day history of worsening shortness of breath and a one-week history of dry cough, fatigue, diffuse myalgia, and lightheadedness with a history of COVID-19 sick contacts. On admission, he was noted to be tachycardic with a heart rate of 110 beats/minute and in respiratory distress with a respiratory rate of 22 cycles/minute, with noted use of accessory muscles of respiration and oxygen saturation (SpO_2_) of 78% on room air, which increased to 92% on 15 L of oxygen on a non-rebreather mask that warranted admission to the intensive care unit and was placed on high-flow nasal cannula (HFNC) with a flow rate of 40 L/minute of oxygen and 80% fractional inspiratory oxygen (FiO_2_) with SpO_2_ of greater than 92%.

**Figure 1:**
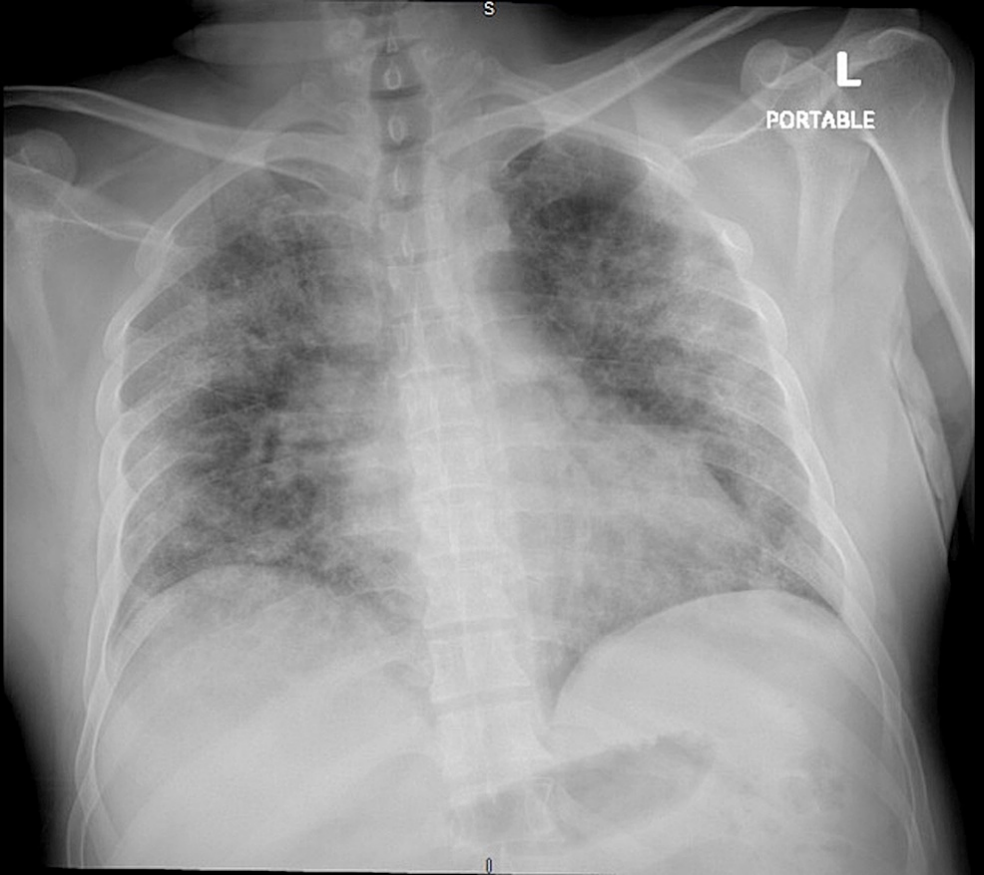
CXR showing bilateral reticular opacities consistent with multifocal pneumonia CXR, chest X-ray

Laboratory investigations were significant for leukocytosis with a white blood cell count (WBC) of 27.74 x 10^3^/mcL, neutrophilia of 84.8%, lymphopenia of 7.6%, thrombocytopenia with platelets of 148 x 10^3^/mcL, mild transaminitis with aspartate aminotransferase of 41U/L and alanine transaminase of 48U/L on hepatic function panel, international normalized ratio of 1.3, D-dimer elevated at 19,898 ng/mL DDU, and lactate dehydrogenase elevated at 1,036 U/L; other laboratory investigations are mentioned in Tables 1, 2. COVID-19 polymerase chain reaction (PCR) test was positive, which confirmed COVID-19 infection. Chest X-ray showed characteristics of typical COVID-19 pneumonia with bilateral patchy reticular interstitial opacities (Figure 1).

**Table 1:** Complete blood count at the time of presentation WBC, white blood cell

Variable	Value at presentation	Reference range
WBC	27.74	4.80–10.80 x 10^3^/mcL
Hemoglobin	17.4	14.0–18.0 g/dL
Platelets	148	150–450 x 10^3^/mcL
Neutrophils	84.8	44.0–70.0%
Lymphocytes	7.6	20.0–45.0%
Monocyte	3.1	2.0–10.0%

**Table 2:** Comprehensive metabolic panel WBC, white blood cell; CO_2_, carbon dioxide; BUN, blood urea nitrogen; AST, aspartate transaminase; ALT, alanine transaminase

Variable	Value at presentation	Reference range
Sodium	137	136–145 mmol/L
Potassium	4.7	3.5–5.1 mmol/L
Chloride	99	98–107 mmol/L
CO_2_	25	22-29 mmol/L
Glucose	125	74–109 mg/dL
BUN	13	7–18 mg/dL
Creatinine	0.7	0.7–1.2 mg/dL
Total protein	7.2	6.4–8.3 g/dL
Albumin	3.30	3.97–4.94 g/dL
Alkaline phosphate	120	40–129 U/L
AST	41	≤40 U/L
ALT	48	≤41U/L
Calcium	8	8.5–10.1 mg/dL
C-reactive protein	29.86	0.00–0.40 mg/dL
Ferritin	375	30–400 nl/ml
Lactate dehydrogenase	1,036	135–225 U/L

The patient was in the intensive care unit for the management of acute severe hypoxemic respiratory failure secondary to viral pneumonia from COVID-19 during the early pandemic when hydroxychloroquine (HCQ) was in a trial phase due to which he was commenced on HCQ along with ceftriaxone and doxycycline to cover for community-acquired pneumonia due to underlying leukocytosis with left shift. He was also placed on therapeutic enoxaparin at 1 mg/kg 12 hourly after a left popliteal vein thrombosis was confirmed by a routine bedside POCUS on day 2. The patient remained on HFNC with oxygen supplementation flow rate at 60 L/minute and FiO_2_ at 80 % until day 4. POCUS on day 4 showed normal left ventricular (LV) function with no pericardial effusion and diffuse B lines bilateral lung fields anteriorly, and the patient’s oxygen requirement worsened with subsequent intubation with maximum ventilator setting of positive end-expiratory pressure (PEEP) of 10, FiO_2_ of 100%, tidal volume of 370 mL, respiratory rate of 22 cycles/minute, and SpO_2_ > 92%.

**Figure 2:**
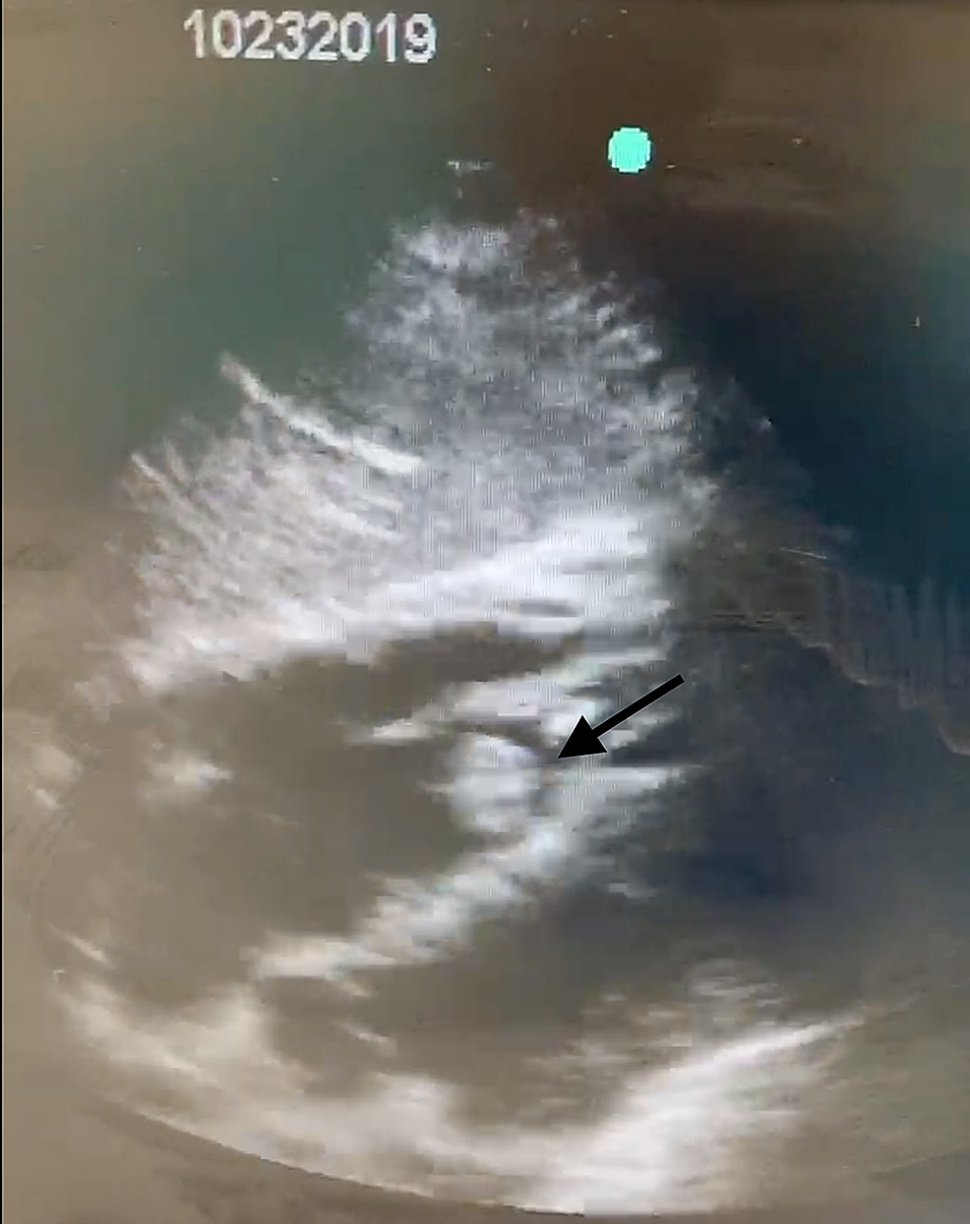
Point-of-care ultrasound of the heart showing thrombus in transit in the right ventricle (black arrow).

POCUS on day 5 (Figure 2) showed moderately decreased LV function and signs of right ventricular volume and pressure overload with cardiac thrombus visualized in the right ventricle, which was considered to be contributing to the clinical refractory hypoxia and newly developing shock, for which the patient received full-dose systemic thrombolysis dosed at 100 mg intravenous infusion of alteplase over two hours along with the commencement of nitric oxide to enhance pulmonary vasodilation for better oxygenation with continued maximum ventilatory support of PEEP of 10 and FiO_2_ 100%.

After the full-dose systemic thrombolysis for massive PE, the patient’s oxygen requirement reduced markedly and was gradually weaned off the nitric oxide and the ventilator and was extubated, with subsequent POCUS showing return of the normal right ventricular function and disappearance of cardiac thrombus. The patient was also monitored for signs of minor and major bleeding over 48 hours. Trending post-thrombolysis d-dimer levels showed marked reduction from the levels at the time of admission (Table 3). Hemoglobin remained stable with no obvious bleeding reported within 48 hours after alteplase infusion. The patient was placed on long-term direct-acting oral anticoagulant rivaroxaban at the time of discharge for provoked VTE.

**Table 3:** Trend of D-dimer values before anticoagulation and after anticoagulation

Component	Day 1	Day 5	Day 5	Day 8	Day 30
	On admission before anticoagulation	After anticoagulation			
D-dimer quantitative, 0–243 ng/mL DDU	19,898 (H)	>3,680 (H)	2,924	1,902	265

## Discussion

COVID-19 is primarily a respiratory infection caused by the severe acute respiratory syndrome coronavirus 2 (SARS-CoV-2), which is known to have a predilection to widespread venous and arterial thrombosis due to endothelial dysfunction, activation of platelets, systemic inflammatory response, hypoxia, and stasis [7,10]. The exact pathophysiology behind the hypercoagulable state remains unknown. However, it has been reported that physiological response may be enhanced by accelerated coagulation seen in response to several infections [11-13]. Coagulation also confers defense against severe infections due to its special immune response function [14]. However, the notable pattern of numerous venous thromboembolic events reported calls for being on the lookout, which would enable timely appropriate management [5-7].

COVID-19 is known to cause both micro- and macrovascular thrombosis [15-18]. Whereas, some post-mortem studies have also reported the role of hypercoagulability in patients who die from COVID-19 by discovering underlying deep vein thrombosis and microthrombi in alveolar capillaries [19,20]. Incidents of clinical deterioration and need for mechanical ventilation resulting in intensive care admissions and exceeding mortality are correlated with D-dimer elevation, which is known to cause hypercoagulable state and adverse thromboembolic events [10].

No routine investigation for acute VTE is currently recommended with elevated D-dimer values, but it is useful in the risk stratification of patients and to place them on either prophylactic or therapeutic doses of anticoagulation based on the D-dimer cut off indicator, which is determined institutionally [10,21-23]. The reported number of events with VTE and refractory hypoxia remains high despite being maintained on prophylactic or therapeutic doses of anticoagulation in patients with a high clinical indication, which has shown a reduction in mortality otherwise [5,6].

Therefore, VTE should be a prioritized differential diagnosis in a COVID-19 patient with typical clinical symptoms of deep vein thrombosis, discordant hypoxia to known respiratory pathologies, or unexplained right ventricular dysfunction with cardiogenic shock occurring acutely. A case series of three patients with COVID-19 and acute respiratory distress syndrome related respiratory failure recently reported transient improvements in oxygenation after receiving alteplase 50 mg (25 mg bolus followed by 25 mg IV over two hours) [24]. At present, there is no high-impact evidence for using systemic thrombolysis due to the risk of adverse events being high.

Also, a challenge to conduct diagnostic imaging studies to detect deep venous thrombosis or pulmonary embolism (PE) in COVID-19 patients is the transmission of disease to other patients or healthcare workers [25]. However, it is also important to safeguard the access to available technology to care for the common non-COVID-19 afflicted patient population having a thrombotic disease. A novel solution to this challenge may be the use of POCUS to look for signs of potentially developing DVT, newly occurring dysfunction of the right ventricle, and in rare instances of a clot in transit [6,9,13].

## Conclusions

COVID-19 is predominantly a pulmonary disease with underlying systemic involvement associated with widespread vascular thrombosis and thromboembolic events. We present a classic case of VTE in a patient on therapeutic enoxaparin for venous thrombosis developing refractory hypoxia with a clot in transit detected in the right ventricle using POCUS. The patient was subsequently treated with a systemic thrombolysis, resulting in a good outcome. Although COVID-19 pneumonia patients are being maintained on therapeutic anticoagulants if coexisting thrombosis or elevated d-dimer levels are detected, recognizing the events of VTE in the critically ill that can cause rapid deterioration and refractory hypoxia and commencement of systemic thrombolysis can result in the reduction of mortality.

We also emphasize the importance of the use of POCUS in such cases of refractory hypoxia to aid management and improve clinical outcomes. Further studies are required to establish the importance of consideration of systemic thrombolysis in the presence of clinical indication, such as thrombus in transit with impending PE, highly likely PE with hemodynamic compromise, ST-elevation myocardial infarction, acute ischemic stroke.
